# Toward Regulatory Effects of Curcumin on Transforming Growth Factor-Beta Across Different Diseases: A Review

**DOI:** 10.3389/fphar.2020.585413

**Published:** 2020-12-14

**Authors:** Milad Ashrafizadeh, Ali Zarrabi, Kiavash Hushmandi, Vahideh Zarrin, Ebrahim Rahmani Moghadam, Farid Hashemi, Pooyan Makvandi, Saeed Samarghandian, Haroon Khan, Fardin Hashemi, Masoud Najafi, Hamed Mirzaei

**Affiliations:** ^1^ Faculty of Engineering and Natural Sciences, Sabanci University, Orta Mahalle, Istanbul, Turkey; ^2^ Sabanci University Nanotechnology Research and Application Center (SUNUM), Istanbul, Turkey; ^3^ Department of Food Hygiene and Quality Control, Faculty of Veterinary Medicine, University of Tehran, Tehran, Iran; ^4^ Laboratory for Stem Cell Research, Shiraz University of Medical Sciences, Shiraz, Iran; ^5^ Department of Anatomical Sciences, School of Medicine, Student Research Committee, Shiraz University of Medical Sciences, Shiraz, Iran; ^6^ Islamic Azad University, Kazeroon, Iran; ^7^ Centre for Micro-BioRobotics, Istituto Italiano di Tecnologia, Pisa, Italy; ^8^ Department of Basic Medical Sciences, Neyshabur University of Medical Sciences, Neyshabur, Iran; ^9^ Department of Pharmacy, Abdul Wali Khan University, Mardan, Pakistan; ^10^ Student Research Committee, Department of Physiotherapy, Faculty of Rehabilitation, Ahvaz Jundishapur University of Medical Sciences, Ahvaz, Iran; ^11^ Medical Technology Research Center, Kermanshah University of Medical Sciences, Kermanshah, Iran; ^12^ Radiology and Nuclear Medicine Department, School of Paramedical Sciences, Kermanshah University of Medical Sciences, Kermanshah, Iran; ^13^ Research Center for Biochemistry and Nutrition in Metabolic Diseases, Institute for Basic Sciences, Kashan University of Medical Sciences, Kashan, Iran

**Keywords:** curcumin, transforming growth factor-β, diseases, antioxiants, natural compound

## Abstract

Immune response, proliferation, migration and angiogenesis are juts a few of cellular events that are regulated by transforming growth factor-β (TGF-β) in cells. A number of studies have documented that TGF-β undergoes abnormal expression in different diseases, e.g., diabetes, cancer, fibrosis, asthma, arthritis, among others. This has led to great fascination into this signaling pathway and developing agents with modulatory impact on TGF-β. Curcumin, a natural-based compound, is obtained from rhizome and roots of turmeric plant. It has a number of pharmacological activities including antioxidant, anti-inflammatory, anti-tumor, anti-diabetes and so on. Noteworthy, it has been demonstrated that curcumin affects different molecular signaling pathways such as Wnt/β-catenin, Nrf2, AMPK, mitogen-activated protein kinase and so on. In the present review, we evaluate the potential of curcumin in regulation of TGF-β signaling pathway to corelate it with therapeutic impacts of curcumin. By modulation of TGF-β (both upregulation and down-regulation), curcumin ameliorates fibrosis, neurological disorders, liver disease, diabetes and asthma. Besides, curcumin targets TGF-β signaling pathway which is capable of suppressing proliferation of tumor cells and invading cancer cells.

## Introduction

Thanks to the previously conducted research over past decades to assist scientists in comprehensive understanding of molecular signaling pathways and mechanisms, and how to deal with them in different diseases and disorders (Farooqi et al., 2020). Interdisciplinary research and the emergence of cutting-edge technologies have made it possible to look for major signaling pathways and their regulation (Farooqi et al., 2019a). There has been an explosion in the field of molecular biology and recently published articles have also confirmed this fact (Farooqi et al., 2019b; Fayyaz et al., 2019). Along with identification of molecular pathways, scientists have tried to develop synthetic drugs in their regulation. It is worth mentioning that plus to synthetic drugs, there has been a great trend toward plant derived-natural compounds in the regulation of molecular pathways and mechanisms (Najafi et al., 2019b; Mortezaee et al., 2020). Nowadays, researchers are more interested in naturally occurring compounds compared to synthetic therapeutics. This emanates from the fact that synthetic drugs are designed for just a purpose (for instance, treatment of a particular disease) and targeting a certain pathway and mechanism, while a large number of studies have revealed that naturally-occuring compounds are capable of affecting a wide variety of molecular signaling pathways (Guo et al., 2019; Mortezaee et al., 2019a; Mortezaee et al., 2019b; Mortezaee et al., 2019c). This multi-targeting nature of herbal compounds have attracted much fascination. In addition to multi-targeting property, it has been demonstrated that herbal compounds have minimal toxicity or even lack toxicity against normal cells (Farhood et al., 2019a; Farhood et al., 2019b), whereas synthetic drugs negatively affect organs of body. For instance, such story is obviously observed in chemotherapy. It seems that synthetic drugs applied in chemotherapy have a number of adverse effects against normal cells and may induce renotoxicity, hepatotoxicity and so on, while plant derived-natural compounds can be used as potential chemotherapeutic agents with negligible side effects (Najafi et al., 2019a). All of these statements advocate from the fact that herbal compounds are efficient agents in treatment of diseases and they can be applied to target various molecular pathways. In the current mechanistic review, we specifically discuss the potential of curcumin in targeting transforming growth factor-β (TGF-β) in disease therapy to direct further studies for research in this field.

## Curcumin: An Overview of the Pharmacological Impacts and Limitation

**FIGURE 1:**
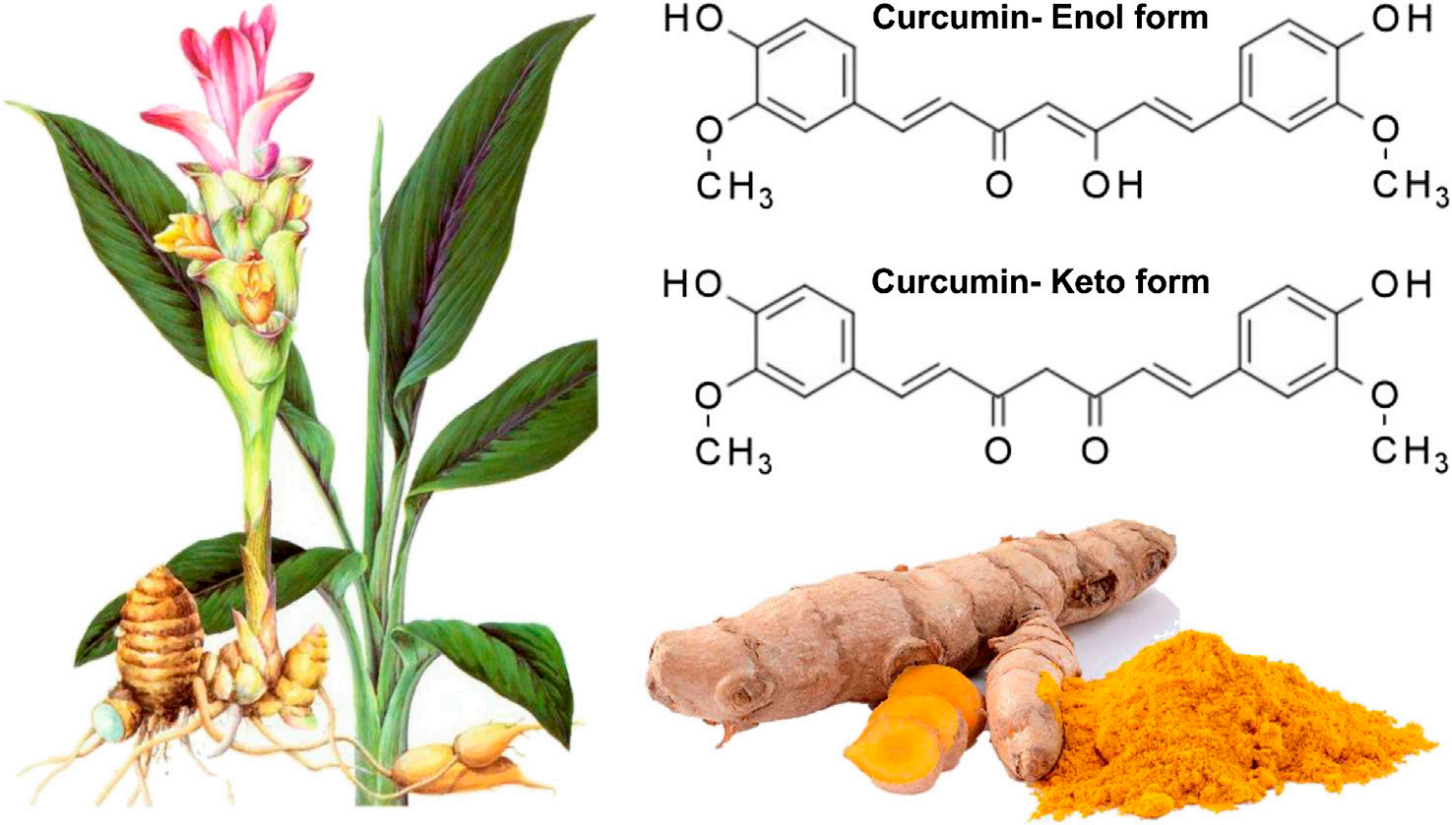
The Structure of curcumin in which its enolic exsits organic solvents whereas its keto form presence in aquoues mwedia (Zheng et al., 2018). Reprinted with permission from the publishers.

Curcumin, a phenolic compound, is also known as diferuloylmethane with chemical name of (1E, 6E)-1,7-bis(4-hydroxy-3-methoxyphenyl)hepta-1,6-diene-3,5-dione) (Figure 1) (Baldi et al., 2020; Chainoglou and Hadjipavlou-Litina, 2020; Salehi et al., 2020a; Stohs et al., 2020). This biologically active compound occurs in high amounts in rhizome and roots of turmeric plant (*Curcuma longa*) (Hesari et al., 2019). Apart from curcumin, there are also two other curcuminoids in this plant including demethoxycurcumin (DMC) and bis-demethoxycurcumin (BDMC). It has been demonstrated that curcuminoids comprise 2–4% of dry turmeric root powder (Ak and Gülçin, 2008; Prasad et al., 2014; Amalraj et al., 2017; Kocaadam and Şanlier, 2017; Kunnumakkara et al., 2017; Rahmani et al., 2018; Yeung et al., 2019). Curcumin has a yellow color and can be used in several applications, e.g., as a food flavoring and coloring agent, and herbal nutrition supplement (Aggarwal et al., 2006; Aggarwal et al., 2007). Curcumin was isolated for the first time at the impure form in 1815, but Lampe and colleagues characterized curcumin in 1910 in term of structure and chemically synthesized it (Lampe and Milobedzka, 1913; Gupta et al., 2012; Mehanny et al., 2016). As a bis-α,β-unsaturated *β*-diketone, curcumin exhibits keto-enol tautomerism. The enol form of curcumin is widely found in alkaline solutions, while its keto form is prevalent in acidic and natural pH (Sharma et al., 2005). It is worth mentioning that curcumin is used in food, pharmaceutical and textile industries (Aggarwal and Harikumar, 2009). It seems that curcumin has been common for treatment of diseases in Asia, particularly traditional Indian medicine and this returns back to 2,500 years ago (Unlu et al., 2016). The curcumin has been considered as an efficient agent in treatment of different ailments such as infection therapy in eye and skin diseases, rheumatism, dyspepsia, and irritable bowel disease, amonng others (Singh, 2007; Hatcher et al., 2008; Qin et al., 2009; Lucariello et al., 2015; Zhou et al., 2017a; Perna et al., 2018; Esposito et al., 2019; Hay et al., 2019).

The first experiment related to therapeutic impact of curcumin was published in 1937 (Zhou et al., 2011), and since then, much attention has been directed toward revealing the extraordinary pharmacological activities of curcumin. It has been reported that curcumin has valuable therapeutic and biological activities such as antioxidant (Kharat et al., 2020), anti-inflammatory (Sneharani, 2019), anti-diabetic (Xia et al., 2020), hepatoprotective (Dogaru et al., 2020), cardioprotective (Hadi et al., 2019; Hallajzadeh et al., 2019; Kuszewski et al., 2019), neuroprotective (Sturzu et al., 2019), anti-microbial (Rai et al., 2020), anti-tumor (Bahrami et al., 2019; Shabaninejad et al., 2020; Weng and Goel, 2020) and improving dyslipidemia (Roxo et al., 2019) and ischemic-reperfusion (Ahmed et al., 2019). Curcumin possesses great solubility in oil-based solutions. Besides, being insoluble in water at acidic and neutral pH, curcumin is soluble at alkaline pH. As mentioned earlier, in spite of excellent therapeutic activities, a variety of issues have remarkably restricted the effectiveness of curcumin. The most important hurdle is its poor solubility in aqoues media (11 ng/ml) as well as its rapid metabolism into an inactive metabolite (Valencia et al., 2019; Moeini et al., 2020). . In light of this, a number of research have been devoted on the solubility enhancements or encapsulation of curcumin targeted drug delivery platforms for biomedical applications (Deljoo et al., 2019; Zare et al., 2019a; Zare et al., 2019b). For instance, a number of nanoscale carriers (e.g., micelles, liposomes, polymeric nanocarriers, lipid nanoparticles, and carbon nanotubes) have been developed to encapsulate hydrophobic active compounds such as curcumin (Bian and Guo, 2020; Li et al., 2020b; Hu et al., 2020; Varshosaz et al., 2020; Zhao et al., 2020). Such platforms enhanced the therapeutic efficacy of curcumin along with prolonged delivery. In the following section, the influence of curcumin on molecular pathways in exerting its pharmacological activities is highlighted.

## Curcumin and Molecular Pathways and Mechanisms

Notably, curcumin is suggested to affect various molecular signaling pathways and mechanisms (Ghasemi et al., 2019; Bagherian et al., 2020; Mardani et al., 2020; Salehi et al., 2020b). Until now, no naturally occurring compound has been investigated similar to curcumin. Herein, the potential therapeutic impacts of curcumin mediated by its effect on molecular pathways and mechanisms are discussed. The nuclear factor erythroid 2-related factor 2 (Nrf2) is well-known due to its capability in improving antioxidant defense system via targeting down-stream mediators including heme oxygenease-1 (HO-1), superoxide dismutase (SOD) and NADPH quinone reductase 1 (NQO1) (Song et al., 2020). It is said that antioxidant activity of curcumin is mainly mediated by stimulation of Nrf2 signaling pathway (Zhang et al., 2020a). Multiple studies have investigated the potential of curcumin in diabetes mellitus (DM) treatment, as a chronic metabolic disorder (Funamoto et al., 2019). Mechanistically, curcumin improves insulin resistance and dyslipidemia, and remarkably diminishes levels of glucose via upregulation of GLUT1 and GLUT4 (Al-Saud, 2020). The calcified aortic valve disease (CAVD) is a primary valve disease that negatively affects a high number of people worldwide (Kirchhof et al., 2016). A variety of factors are involved in CAVD development, but it appears that inflammatory factors play a pivotal role (Lee and Choi, 2018). The nuclear factor-kappaB (NF-κB) is suggested to induce inflammation (Sinjari et al., 2019). The administration of curcumin effectively suppresses the progression and development of CAVD via down-regulation of NF-κB and inhibiting its nuclear translocation (Zhou et al., 2020). Two factors are vital in amelioration of damages on cells and improving a disease that include reducing stress and inhibition of apoptotic cell death. In attenuation of diabetic nephropathy, curcumin diminishes apoptosis via down-regulation of pro-apoptotic factors Bax and caspase-3, while it induces autophagy through upregulation of Beclin-1 and ATG5, resulting in reduced cell stress (Zhang et al., 2020c). It is worth mentioning that curcumin induces apoptotic- and autophagic-cell death in cancer therapy. However, it is held that autophagy can determine the number of cancer cells undergoing apoptosis, so that cancer cells with high autophagy influx demonstrate a relative resistance into apoptosis (Lee et al., 2020a). Apoptosis can be triggered by endoplasmic reticulum (ER) stress in which glucose-regulated protein 78 (GRP78), CCAAT-enhancer-binding protein homologous protein (CHOP) and activating transcription factor 4 (ATF4) are induced by unfold protein response (UPR) to ameliorate ER stress by stimulation of apoptosis (Di Conza and Ho, 2020). Curcumin stimulates neuroprotective effects by down-regulation of GRP78 and ATF4 to attenuate ER stress-mediated apoptosis in neuronal cells (Keshk et al., 2020). Noteworthy, curcumin-mediated Notch upregulation protects neuronal cells against cytotoxic agents such as bisphenol A (BPA) (Tandon et al., 2020). Taking everything into account, based on the recently published articles, it can be said that curcumin is a potential naturally occurring compound in treatment of various disorders and diseases. This is due to capability of curcumin in affecting a variety of molecular signaling pathways and mechanisms that are discussed in this section (Liczbiński et al., 2020; Mohajeri et al., 2020; Sharifi et al., 2020).

## Transforming Growth Factor-Beta Signaling Pathway: From Basics to Role in Pathological Events

The TGF-β is a dynamic and sophisticated molecular signaling pathway with pleiotropic impacts that modulate various biological mechanisms such as cell proliferation, cell differentiation, angiogenesis, motility, invasion, and immune response (Bai et al., 2019; Boguslawska et al., 2019; Finnson et al., 2019; Soleimani et al., 2019; Lai et al., 2020; Li and Wu, 2020; Lin and Wu, 2020; Tzavlaki and Moustakas, 2020). The TGF-β family possesses 33 genes that are capable of encoding homodimeric or heterodimeric secreted cytokines (Heldin and Moustakas, 2016; Derynck and Budi, 2019). Then, these proteins are cleaved via secretory pathway to produce mature dimeric ligands (Ten Dijke and Arthur, 2007; Heldin and Moustakas, 2016). The ribosomes present on the rough ER participate in synthesis of TGF-β and then, other processes including eliminating *N*-terminal signal peptide, protein folding and glycosylation occur in their route of ER into Golgi apparatus (Manning et al., 2002; Ten Dijke and Arthur, 2007). The TGF-β protein folding relies on formation of intermolecular disulfide bonds in *N*- and C-terminal region (Ten Dijke and Arthur, 2007). The glycosidation of *N*-terminal segment of TGF-β leads to the inactivation of TGF-β (Miyazono and Heldin, 1989), showing that further process is needed to activate TGF-β. Next, proteolytic cleavage of disulfide bonds by furin family proteins result in generation of two characteristic proteins including *N*-terminal long dimeric and disulfide-linked propeptide, known as latency-associated peptide (LAP) and C-terminal short dimeric disulfide-linked polypeptide, known as mature TGF-β (Ten Dijke and Arthur, 2007; Derynck and Budi, 2019). The signaling pathway of TGF-β is of interest and includes canonical and non-canonical pathways.

### Canonical Pathway

The transmembrane serine/threonine kinases such as TGF-β type II (TβRII) and type I (ALK5) are involved in canonical pathway of TGF-β signaling (Wrighton et al., 2009; Liu and Feng, 2010). The TGF-β induces phosphorylation of ALK5 by binding into TβRII (Massagué, 1998; Wrighton et al., 2009; Ahmadi et al., 2019). Then, Smad cascade is activated, so that ALK5 stimulates phosphorylation of Smad2 and Smad3 proteins to form a complex with Smad4. This complex translocates and accumulates in nucleus to regulate gene expression by cooperation with co-activators such as CBP/p300 and co-repressors such as tumor growth-interacting factor (TGIF), Ski and SnoN (Schmierer and Hill, 2007; Hill, 2009). Notably, there are a number of Smads, known as inhibitor Smads (I-Smads) that include Smad6 and Smad7. They are able to inhibit TGF-β signaling pathway by acting as an antagonist. The Smad6 is suggested to suppress Smad1 by competing with Smad4 for binding into phosphorylated Smad1 (Hata et al., 1998). The Smad7 forms a negative feedback loop with TGF-β signaling (Yan et al., 2009). The Smad7 suppresses TGF-β signaling by competing with Smad2/3 for binding into ALK5 (Yan et al., 2009).

### Non-Canonical Pathway

**FIGURE 2:**
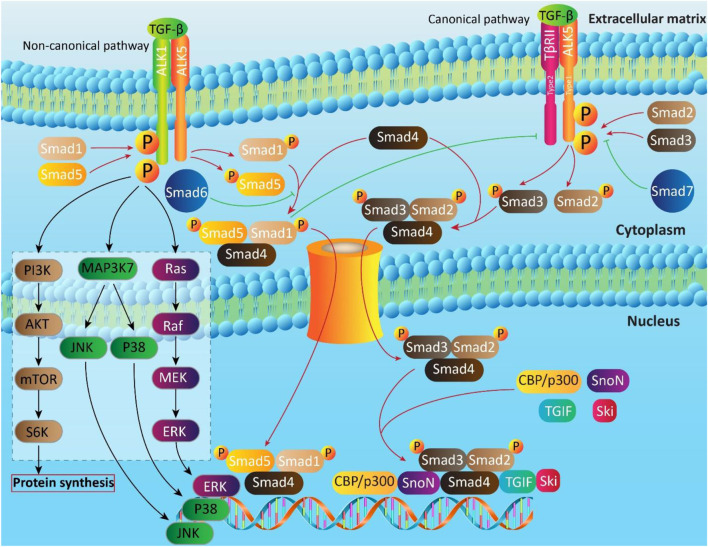
The canonical and non-canonical pathways of TGF-β signaling. Smads are key executers in TGF-b signaling that regulate expression of target genes by translocating into nucleus. In non-canonical pathway, in addition to Smads, other moleculra pathways such as PI3K, MAPK and Ras are involved.

The most important axis in non-canonical pathway of TGF-β is ALK1-Smad1/5. In fact, ALK1 as a TGF-β type I receptor, plays a key role in non-canonical pathway of TGF-β and is expressed in chondrocytes, endothelial cells and so on. Upon TGF-β attachment, ALK1 is induced to form a complex with ALK5, resulting in Smad1/5 activation (Finnson et al., 2008; Finnson et al., 2010; Pardali et al., 2010; van der Kraan et al., 2010; Farhood et al., 2020), and suppressing ALK5-Smad2/3 signaling (Finnson et al., 2008). The mitogen-activated protein kinase (MAPK), phosphatidylinositol 3-kinase (PI3K) and Rho-like GTPase contribute to non-canonical pathway of TGF-β (Figure 2) (Derynck and Zhang, 2003; Moustakas and Heldin, 2005; Zhang, 2009).

### The Role of Transforming Growth Factor-Beta in Pathological Events

Multiple studies have evaluated the role of TGF-β signaling pathway in different malignancies and disorders. It is not possible to comprehensively discuss the role of TGF-β signaling in diseases in this article (limitation of space) and we cite some of the great reviews for further information (Fionda et al., 2020; Munoz et al., 2020; Regis et al., 2020). However, we briefly describe the role of TGF-β signaling pathway in pathological events to pave the road for discussing the relationship between curcumin and TGF-β in different diseases.

Based on the recently published articles, it seems that enhanced activity of TGF-β predisposes muscle into damage. The TGF-β elevates the levels of fibroadipogenic progenitors (FAPs) to induce fibro-calcification of muscle, resulting in muscle degeneration and inhibition of regenerative myogenesis (Mazala et al., 2020). In respect to the role of TGF-β in degeneration, studies have focused on regulation of TGF-β signaling in disease therapy. It appears that administration of ginsenoside Rg1 remarkably diminishes airway collagen volume fraction, decreases the levels of inflammatory cytokines, and ameliorates pulmonary fraction. The examination of molecular pathways demonstrates that down-regulation of TGF-β1/Smad3 axis mediates antifibrotic impact of this compound (Guan et al., 2020). In fact, these studies confirm the pro-inflammatory role of TGF-β, and its association with organ damage. Compounds similar to ginsenoside Rg1 with inhibitory effects on TGF-β can be beneficial in preventing TGF-β-mediated organ damage. As pulmonary fibrosis is going to be an increasing concern, researchers have focused on finding both pharmacological and genetic interventions for this disorder. It is suggested that tripartite motif-containing 33 (TRIM33) alleviates pulmonary fibrosis via inhibition of TGF-β signaling pathway (Boutanquoi et al., 2020). This study demonstrates that upstream molecular pathways of TGF-β can be targeted in disease therapy.

As it was mentioned earlier, DM is a chronic metabolic disorder with high incidence rate, demanding novel strategies in its treatment and management. The *β*-cell dysfunction is a risk factor of DM. So, protection of *β*-cells is of considerable importance in DM therapy. It has been reported that TGF-β/Smad3 contributes to apoptotic cell death in *β*-cells, leading to their dysfunction and glucose tolerance (Lee et al., 2020b). Although upstream mediators are able to affect TGF-β in pathological events, increasing evidence exhibits that TGF-β signaling pathway can induce fibrosis and tracheal stenosis by stimulation of down-stream fibrotic mediators PI3K/Akt (Xiao et al., 2020). By inhibition of TGF-β, a decrease occurs in levels of inflammatory factors such as ILs and TNF-α to attenuate inflammatory diseases (Liu et al., 2020b). The TGF-β induces impairments in airway via enhancing cell migration and extracellular matrix (ECM) production, and by inhibition of TGF-β, the aforementioned mechanisms undergo down-regulation (Kim et al., 2020). It is worth mentioning that microRNAs (miRs) can function as upstream regulators of TGF-β. In stimulation of cardiac fibrosis, miR-21 activates TGF-β/Smad3 axis, while it decreases expression of Smad7, as an I-Smad (Yang et al., 2020).

The epithelial-to-mesenchymal transition (EMT) is a vital mechanism for inflammation, metastasis of cancer cells and fibrosis (Gui et al., 2020; Lee et al., 2020d). The TGF-β is able to stimulate EMT via upregulation of Smad2/3 (Fang et al., 2020). So, suppressing TGF-β can ameliorate inflammation and fibrosis in lung epithelial cells. It has been reported that TGF-β alleviates development of ovarian hyperstimulation syndrome via VEGF overexpression (Wan et al., 2020). These studies obviously demonstrate the role of TGF-β in diseases. Noteworthy, TGF-β plays a significant role in cancer progression. In enhancing migration and metastasis of cancer cells, TGF-β induces EMT through Smad4 upregulation (Xiong et al., 2020). Upstream oncogenic factors such as HOXD9 stimulate expression of TGF-β, leading to enhanced proliferation and growth of tumor cells (Wardhani et al., 2020). Notably, TGF-β overexpression induces resistance of cancer cells into chemotherapy (Qin et al., 2020). Consequently, studies have focused on inhibition of TGF-β signaling pathway in cancer therapy. It is held that down-regulation of TGF-β sensitizes cancer cells into anti-tumor immunity and remarkably diminishes their growth and proliferation (Horn et al., 2020).

These studies highlight the ponteitla contribution of TGF-β signaling in disease development. Notworthy, a variety of down-stream and upstream mediators of TGF-β exist that mediate its role in pathological events. Even molecular mechanisms are down-stream targets of TGF-β. For instance, DM treatment and preventing apoptosis in *β*-cells are performed via down-regulation of TGF-β. In fact, TGF-β induces Smad3 to trigger apoptosis in *β*-cells, providing condition for DM emergence (Lee et al., 2020c). Autophagy is another type of programmed cell death that can be affected by TGF-β. This molecular pathway is able to dually down-regulate/upregulate autophagy in normal and cancerous cells (Mao et al., 2019; Jin et al., 2020). So, revealing interaction between TGF-β and autophagy can be of importance for developing therapeutics. The interesting point is that TGF-β can affect various molecular pathways in disease development including mTOR (Chen et al., 2020c), PI3K/Akt (Wang et al., 2020a), Wnt (Liu et al., 2019), and STAT3 (Dees et al., 2020). There are also molecular pathways that can function as upstream mediators of TGF-β in pathological events. MiRs (Ge et al., 2019), lncRNAs (Tang et al., 2019), circRNAs (Bai et al., 2020), TRPM2 (Wang et al., 2019b) and so on can regulate TGF-β signaling in different disorders. Pharmacological or genetic interventions of aforementioned signaling networks can pave the road into effective treatment of diseases.

Most of the experiments are in line with pro-inflammatory role of TGF-β in diseases. This pro-inflammatory role is in favor of disease development and progression. So, down-regulation of TGF-β is advantageous in suppressing inflammation-mediated disease development. For instance, TGF-β induces inflammation to enhance progression and aggressive behavior of hepatocellular carcinoma cells. Expression of TGF-β is positively affected by Dickkopf-1 (DKK1) (Fezza et al., 2019). Hence, inhibiting DKK1/TGF-β axis can lead to preventing inflammation-mediated cancer growth. ILs with anti-inflammatory roles down-regulate expression of TGF-β in suppressing inflammation. IL-22 is able to inhibit TGF-β signaling via Notch1 inhibition, leading to a decrease in inflammation and renal fibrosis (Tang et al., 2020). Although these studies demonstrate pro-inflammatory role of TGF-β, it appears that TGF-β also possesses anti-inflammatory roles. Theacrine, as an anti-inflammatory compound, prevents synovial hyperplasia and inflammatory cell infiltration in joint tissues. This anti-inflammatory effect is mediated via TGF-β induction and subsequent upregulation of Smad expression (Gao et al., 2020b). In the next sections, we investigate relationship between curcumin and TGF-β in different diseases.

## Curcumin and Transforming Growth Factor-Beta in Different Diseases

### Liver Diseases

The incidence of liver diseases has undergone an increase due to lifestyle. They are commonly occur in patients who suffer from obesity and alcohol abuse (Buonomo et al., 2019). The liver fibrosis is a chronic liver disease in which extracellular proteins such as collagen accumulate. In diseased liver cells, a number of cells including hepatic stellate cells, portal fibroblasts and myofibroblasts produce collagen. It is said that TGF-β1 is involved in stimulation of aforementioned cells (Komolkriengkrai et al., 2019). In rats exposed to carbon tetrachloride (CCl_4_), the chronic liver fibrosis occurs due to enhanced expression of TGF-β1. TGF-β is one of the signaling pathways that is down-regulated by curcumin in alleviation of liver fibrosis (Abo-Zaid et al., 2020). Liver dysfunction is a common phenomenon during DM. It is worth mentioning that TGF-β/Smad signaling pathway can lead to liver injury and inflammation in DM, and its inhibition attenuates liver damage (Zhang et al., 2019a). Incorporation of curcumin into polymeric nanoparticles significantly enhances its bioavailability and therapeutic impacts, leading to amelioration of DM-mediated liver injury by inhibition of TGF-β1 (El-Naggar et al., 2019). The paraquat is a common herbicide commonly applied in different countries. The reports display that 5–15 ml of 20% concentration of paraquat can result in moderate or severe poisoning (Wesseling et al., 2001; Baltazar et al., 2013). Several studies have shown that exposing to paraquat can induce liver injury by decreasing antioxidant capacity and stimulation of inflammation (Gao et al., 2020a; Liu et al., 2020c). Although studies have put much emphasis on the involvement of oxidative stress and inflammation in paraquat-mediated toxicity, the role of TGF-β signaling pathway is uncertain in liver toxicity. A recently published article has shown that in stimulation of liver toxicity, paraquat enhances expression of TGF-β1. The curcumin supplementation attenuates paraquat-mediated liver injury via down-regulation of TGF-β1 signaling pathway (Chen and Fu, 2018).

### Cancer

It has been reported that in addition to epithelial cells, tumor microenvironment (TME) and tumor cell interaction plays a pivotal role in cancer progression (Jung and Le, 2018). The cancer associated fibroblasts (CAFs) are able to induce chemoresistance and ensure cancer progression (Houthuijzen and Jonkers, 2018; Liao et al., 2018). The different factors such as TGF-β, matrix metalloproteinases (MMPs) and so on contribute to cancer initiation via activation of CAFs (Shiga et al., 2015). The *in vitro* and *in vivo* experiments exhibit that curcumin administration is associated with inhibition of CAF-mediated cancer progression via TGF-β1 down-regulation (Jamalzaei et al., 2020). Cancer cells have higher proliferation and migration compared to normal cells. One of the factors involved in motility and metastasis of cancer cells is EMT. A number of structural and molecular alterations occurs during EMT to produce mesenchymal cells from polarized endothelial cells. In contrast to polarized and static epithelial cells, mesenchymal cells have spindle shape and are not polarized, leading to their migration capability (Lu et al., 2020; Xu et al., 2020). The TGF-β is able to enhance invasion and migration of cancer cells via stimulation of EMT (Li et al., 2019). Exposing lung cancer cells into paraquat (PQ) significantly increases their migration and invasion through TGF-β-induced EMT. The administration of curcumin inhibits TGF-β signaling to suppress EMT, leading to preserving E-cadherin and reducing cancer malignancy (Tyagi et al., 2019). Oxaliplatin (OX) is a chemotherapeutic agent for eliminating cancer cells and enhancing overall survival of patients with cancer (Li et al., 2020a). However, resistance of cancer cells has limited its efficacy. EMT is a potential factor in stimulation of chemoresistance via elevating proliferation and invasion of cancer cells (Cao et al., 2020). The TGF-β can induce EMT and its inhibition by miR-145 suppresses malignant behavior of cancer cells (Chen et al., 2020a). Besides, silencing Linc00511 inhibits EMT and metastasis of cancer cells via TGF-β signaling down-regulation (Zhong et al., 2020). The studies demonstrate that EMT regulation by TGF-β can participate in chemoresistance. Curcumin is able to inhibit nuclear translocation of Smad2/3 via suppressing TGF-β signaling pathway. This leads to a decrease in migration and proliferation of cancer cells and sensitizes them into OX chemotherapy (Yin et al., 2019). It is worth mentioning that curcumin affects TGF-β signaling pathway in cancer therapy via different pathways. In order to suppress invasion and proliferation of cervical, breast and pancreatic cancer cells, curcumin suppresses TGF-β signaling pathway through interfering with Wnt/β-catenin signaling pathway (Nna et al., 2013; Thacker and Karunagaran, 2015; Wang and Yan, 2016). However, there are controversial data showing that curcumin may stimulate TGF-β signaling pathway in inhibition of colon cancer progression (Ramamoorthi and Sivalingam, 2014). A recently published article has revealed a novel pathway of anti-tumor activity of curcumin. It is said that curcumin exerts anti-metastatic activity in pancreatic cancer cells by inhibiting canonical pathway of TGF-β signaling through androgen-dependent and independent manners (Katta et al., 2019). So, in suppressing malignant behavior of cancer cells, curcumin affects TGF-β signaling pathway via targeting another molecular signaling such as Wnt/β-catenin.

The effect of curcumin on TGF-β1 in cancer therapy is dose- and time-dependent manner (Celik et al., 2018). In suppressing malignant behavior of breast cancer cells, curcumin down-regulates expression of TGF-β1 to inactivate Smad2 and MMP-9 (Mo et al., 2012). Exposing breast cancer cells into TGF-β induces secretion of bone-resorptive peptide parathyroid hormone-related protein (PTHrP). This ensures proliferation and invasion of cancer cells. Administration of curcuminoids (25 and 50 mg/kg) suppresses breast cancer malignancy via inhibiting phosphorylation of Smad2 and Smad3 (Wright et al., 2013). It is worth mentioning that in cancer therapy, curcumin can affect upstream mediators of TGF-β1 signaling pathway. As a negative modulator of TGF-β1 signaling pathway, bone morphogenic protein -7 (BMP-7) undergoes upregulation by curcumin to inhibit TGF-β, leading to anti-metastatic activity of curcumin (Dorai et al., 2014). Curcumin can also suppress metastasis of cancer cells via inhibition of TGF-β1-mediated EMT (Xu et al., 2015). In inhibition of TGF-β1-mediated EMT, curcumin inhibits phosphorylation of Smad2 and Smad3 (Zhang et al., 2016). Another pathway in inhibition of TGF-β-mediated EMT by curcumin is that this plant derived-natural compound suppresses Smad2 phosphorylation, its nuclear translocation and interaction with promoter of Snail (Cao et al., 2017). In addition to TGF-β signaling pathway, curcumin is able to suppress receptors in this pathway. Curcumin and its derivatives inhibit ALK5 to down-regulate migration and invasion of cancer cells (Kandagalla et al., 2017).

### Fibrosis

The endothelial-to-mesenchymal transition (EndMT) is a process in which endothelial cells lose their adhesion and polarity, and obtain mesenchymal phenotype, leading to enhanced cell migration and collagen secretion (Maddaluno et al., 2013). Increasing evidence demonstrates that EndMT is vital for production of myofibroblasts in fibrotic tissues or organs (Zeisberg et al., 2007; Potenta et al., 2008; Zeisberg et al., 2008; Li et al., 2009). It has been reported that TGF-β can act as an upstream modulator in stimulation of EndMT (Hou et al., 2019; Yang et al., 2019b). The enhanced expression of TGF-β1 induces an increase in inflammatory factors and asymmetric dimethylarginine (ADMA), whereas a decrease occurs in Nrf2, dimethyl arginine dimethylaminohydrolase-1 (DDAH1), VE-cadherin, secretion of nitric oxide (NO) and activity of nitric oxide synthase (NOS). These factors provide conditions for endothelial cell fibrosis via EndMT induction. It is said that curcumin supplementation down-regulates expression of TGF-β1 to enhance VE-cadherin, DDAH1 and Nrf2 levels, and diminish MMP-9 and ERK1/2 levels. Consequently, TGF-b-mediated EndMT is inhibited to suppress endothelial cell fibrosis (Chen et al., 2020b). Although chemotherapy is a common way in cancer therapy, studies have demonstrated the high adverse effects of chemotherapeutic agents. Cisplatin (CP) is a potential chemotherapeutic agent with excellent anti-tumor activity. However, accumulating data has shown that CP negatively affects kidney by stimulation of inflammation and oxidative stress (Mahran, 2020; Wang et al., 2020c). It is worth mentioning that TGF-β1 mediates nephrotoxicity of CP (Salem et al., 2018). A newly published article has examined the potential of curcumin in improving CP-mediated nephrotoxicity. It seems that a combination of curcumin and arsenic trioxide (ATO) diminishes side effects of CP on kidney and emergence of fibrosis via down-regulation of TGF-β1 (Maghmomeh et al., 2020). The peritoneal dialysis (PD) is a potential strategy of renal replacement therapy for patients who suffer from end-stage renal disease (ESRD). However, PD fluid is not completely biocompatible and has a variety of issues such as low pH, high glucose and lactate, and can lead to abnormalities of peritoneum (Cho et al., 2014). An increase in glucose levels is associated with inflammation and PD. This is mediated via TGF-β1 upregulation and results in peritoneal EMT (Yang et al., 2017). So, targeting TGF-β1 can ameliorate PD-mediated fibrosis. The administration of curcumin remarkably reduces the expression of TGF-β1 in PD animal models to improve ultrafiltration volume, diminish mass transfer of glucose and fibroproliferative response (Zhao et al., 2019a). It seems that during PD, TGF-β signaling pathway enhances migration and motility of cells via stimulation of EMT. Curcumin supplementation is associated with a decrease in migratory ability of these cells via down-regulation of TGF-β and subsequent inhibition of EMT (Zhao et al., 2019b). As a common phenomenon after joint surgery or longtime immobilization, joint contracture has significant pathological alterations including myofibroblast proliferation and enhanced deposition of ECM (Abdel et al., 2012). The prostaglandin E2 (PGE2) is formed by cyclooxygenase metabolism of arachidonic acid and inhibits the migration and proliferation of myofibroblasts, and ECM accumulation (Elias et al., 1985; Bitterman et al., 1986; Fine et al., 1989; Kolodsick et al., 2003; White et al., 2005; Huang et al., 2009). The hyaluronic acid-curcumin conjugate is beneficial in treatment of joint contracture-mediated fibrosis. It is said that hyaluronic acid-curcumin conjugate induces demethylation of prostaglandin E receptor 2 (PTGER2) to enhance its expression. Consequently, activated PTGER2 inhibits TGF-β signaling pathway to negatively affect migration and proliferation of myofibroblasts, resulting in a diminution in fibrosis (Zhang et al., 2019b).

The idiopathic pulmonary fibrosis (IPF) is a multifactorial disorder with involvement of cigarette smoking, air pollution, genetic predisposition, aging and viral infections (Gao et al., 2011; Mora et al., 2017). This chronic and progressive disorder is a form of idiopathic interstitial pneumonia (Gross and Hunninghake, 2001). The IPF interferes with pulmonary function via stimulation of inflammation (Spagnolo et al., 2015). Agents with inhibitory impact on inflammatory cytokines can attenuate IPF. It is suggested that curcumin administration can down-regulate TGF-β1 expression to partially alleviate IPF (Hu et al., 2018). We mentioned earlier that TGF-β involves in fibrosis via stimulation of EMT mechanism. In fact, TGF-β-mediated EMT occurs in a Smad-dependent manner. It is worth mentioning that TGF-β can activate EMT via Smad-independent manner. The TGF-β is able to induce EMT by stimulation of Akt/mTOR signaling pathway (Lu et al., 2019). It has been demonstrated that agents with inhibitory impact on EMT mechanism such as Dendrobium officinale can inhibit TGF-β/Akt/mTOR pathway (Xing et al., 2018; Luo et al., 2019). This shows a novel signaling pathway of TGF-β in EMT induction. The administration of curcumin ameliorates kidney fibrosis by inhibition of EMT via suppressing TGF-β/Akt/mTOR signaling pathway (Zhu et al., 2016). These studies demonstrate that in inhibition of EMT, curcumin affects various molecular pathways (Zhou et al., 2017b). The increasing evidence exhibits that TGF-β signaling pathway can contribute to pulmonary fibrosis by inducing proliferation of lung fibroblasts and their differentiation into myofibroblasts (Massagué, 1998; Hinz et al., 2007; Horbelt et al., 2012). In treatment of pulmonary fibrosis, curcumin inhibits TGF-β1 signaling pathway to suppress proliferation and differentiation of fibroblasts, leading to amelioration of pulmonary fibrosis (Saidi et al., 2019). The proliferative vitreoretinopathy (PVR) is a wound healing response that may be completed by formation of fibrotic tissues. The migration and differentiation of retinal pigment epithelial (RPE) cells play a significant role in formation of fibrotic tissues during PVR (Dartt et al., 2011; Sadaka and Giuliari, 2012). The *in vitro* and *in vivo* experiments have revealed that TGF-β-mediated EMT contributes to trans-differentiation of PRE cells into fibroblasts (Lee et al., 2007; Ali, 2011). A combination of curcumin and epigallocatechin gallate (EGCG) suppresses TGF-β1/Smad3 to inhibit EMT, leading to alleviation of PVR (Shanmuganathan et al., 2017). A same story occurs in CCl_4_ toxicity. The CCl_4_ is able to induce liver fibrosis via inflammation, oxidative stress and stimulates apoptosis (Liu et al., 2020a; Munakarmi et al., 2020; Zhang et al., 2020b). In inhibition of CCl_4_-mediated liver injury, curcumin down-regulates TGF-β1/Smad3 signaling pathway (Peng et al., 2018). These studies highlight the fact that hepatotoxic agents mainly exert their adverse effects via stimulation of TGF-β signaling pathway, and drugs such as curcumin that have modulatory impact on this pathway, are of importance. The important point is that in enhancing the anti-fibrotic activity of curcumin, nanoparticles are of interest, since they can remarkably enhance the bioavailability and therapeutic effect of curcumin (Charoensuk et al., 2016).

### Neurological Disorders

The multiple sclerosis (MS) is an inflammatory neurological disorder negatively affecting central nervous system (CNS) (Bonetti and Raine, 1997). Due to immune attack, some degrees of axon and myelin degeneration occur in MS patients (Goldenberg, 2012). Inflammatory factors play a significant role in MS progression via stimulation of axon degeneration and neuronal dysfunction (Wujek et al., 2002). Enhancing expression of anti-inflammatory factors such as IL-4, IL-5 and TGF-β is a promising strategy in MS therapy (Soleimani et al., 2014). The administration of curcumin remarkably enhances TGF-β expression to suppress inflammation and progression in experimental encephalomyelitis (EAE) model of MS (Esmaeilzadeh et al., 2019). The spinal cord injury (SCI) is a common phenomenon that can occur after accident. It seems that during SCI, TGF-β-SOX9 signaling pathway activates inflammation factor NF-κB to induce glial scar formation. In respect to anti-inflammatory activity of curcumin, it is able to suppress glial scar formation and attenuate SCI via down-regulation of TGF-β-SOX9 axis and subsequent inhibition of NF-κB (Yuan et al., 2019). Lumbar intervertebral disc degeneration (LIDD) is a chronic and progressive disorder characterized by low back pain (Vieira et al., 2014). In respect to its high incidence rate, finding treatments for LIDD is of importance. Increasing evidence demonstrates that TGF-β has dual role in different disorders, so that it may reduce the number of cells undergoing apoptosis in a certain circumstance, while it may enhance apoptotic cell death (Hu et al., 2017). Curcumin administration alleviates LIDD by inhibition of TGF-β1 and TGF-β2 signaling pathways. In fact, studies are in agreement with neuroprotective impact of curcumin mediated by TGF-β down-regulation. It has been reported that in improving neural functionality, curcumin reduces expression of TGF-β1 and TGF-β2 (Yuan et al., 2015).

### Wound Healing

During wound healing, a variety of cells such as inflammatory cells, fibroblasts, keratinocytes, endothelial cells, and growth factors as well as enzymes are involved (Velnar et al., 2009; Abdel-Ghani et al., 2019; Makvandi et al., 2019). The presence of other diseases such as DM impairs wound healing, demanding novel intervention to improve wound process by enhancing growth factor production, induction of angiogenesis, elevating collagen accumulation and macrophage function (Falanga, 2005; Campos et al., 2008). Multiple studies have evaluated the role of TGF-β during wound healing. It seems that upregulation of TGF-β induces angiogenesis to improve wound healing (Zong et al., 2020). Impairment of TGF-β signaling pathway inhibits adaptive response for tissue repair (Jiang et al., 2020). So, restoring expression of TGF-β is a promising strategy in wound healing. Loading a combination of curcumin and lithospermi vadix extract on nanofibrous scaffolds improve wound healing in DM rats partially by stimulation of TGF-β signaling pathway (Yang et al., 2019a). Increasing evidence demonstrates that expression of TGF-β3 undergoes upregulation in scar-less wound healing (Chen et al., 2005). It is held that TGF-β3 induces Smad2/3 phosphorylation at epidermal cells compared to dermal cells (Bandyopadhyay et al., 2006). The TβRII (involved in canonical pathway of TGF-β signaling) demonstrates differential expression during wound healing. The Smad anchor for receptor activation (SARA) attaches into MAD homolog 2 (MH2) of Smad2/3 to regulate nuclear translocation of Smad in TGF-β signaling pathway (Tsukazaki et al., 1998). In amelioration of acute burn injury and accelerating wound healing, Zno-curcumin nanocomposite loaded in hybrid collagen scaffolds stimulates TGF-β3 signaling pathway by upregulation of TβRII and SARA (Kalirajan and Palanisamy, 2019). Tissue engineering has helped us in accelerating wound healing. Using chitosan- and collagen-scaffold is considered as a promising strategy in wound healing (Ramasamy et al., 2014). It seems that chitosan can facilitate wound healing through ameliorating functions of fibroblasts, macrophages and inflammatory cells (Dai et al., 2009). In order to promote functionality of scaffold, collagen can be synergistically used with chitosan, and then, other agents with capability of improving wound healing can be loaded on this scaffold (Dai et al., 2004; Gopinath et al., 2004; Sionkowska et al., 2004). It was shown that curcumin-nanoparticles (CNs) incorporated in collagen-chitosan scaffold are able to remarkably improve wound healing via inhibition of TGF-β1/Smad7 axis to reduce inflammation and pave the road for wound healing (Rezaii et al., 2019). These examples show the significance of curcumin for soft tissue regeneration.

### Asthma

Asthma is a multifactorial disorder with involvement of inflammation, pulmonary edema, airflow obstruction and environmental factors (Jolliffe et al., 2020). This disorder affects a high number of people worldwide (Engelkes et al., 2020; Shinan-Altman and Katzav, 2020), resulting in much attraction into identification its cause and finding novel treatments. It has been demonstrated that pro-inflammatory and pro-fibrotic factors such as TGF-β and TNF-α play a considerable role in asthma pathogenesis (Janulaityte et al., 2020; Liu and Shang, 2020). Agents with inhibitory impact on the expression and level of TGF-β are of considerable importance in asthma therapy. In respect to excellent anti-inflammatory activity of curcumin, it diminishes expression of TGF-β as a pro-fibrotic cytokine to abate airway inflammation and pulmonary edema (Shahid et al., 2019).

### Arthritis

The rheumatoid arthritis (RA) is a joint swelling abnormality that is characterized with synovial inflammation (Mao et al., 2020). Due to the involvement of inflammatory cytokines in RA, anti-inflammatory agents have been of interest in treatment of this disorder. For instance, Brb is able to inhibit RA development via inhibition of IL-21-mediated proliferation of fibroblast like synoviocytes (Dinesh and Rasool, 2019). A newly published article also demonstrates that curcumin can ameliorate RA by targeting inflammation. As a pro-inflammatory cytokine, the expression of TGF-b undergoes down-regulation in rat exposed to curcumin (200 mg/kg), leading to amelioration of inflammation (Wang et al., 2019a).

### Diabetes

The diabetic cardiomyopathy (DCM) is a major complication of both DM type I (DMI) and DM type II (DMII). It seems that DCM affects 12% of patients with DM and can lead to death (Bugger and Abel, 2014; Lorenzo-Almoros et al., 2017). Interestingly, Janus kinase/signal transducer and activator of transcription (JAK/STAT) is involved in intracellular signaling pathways and mechanisms such as proliferation, differentiation and so on by translocation at the route of cytoplasm to nucleus and affecting down-stream targets (Losuwannarak et al., 2019; Wang et al., 2020b). The JAK/STAT signaling pathway can participate in inflammation via stimulation of TGF-β1 (Boengler et al., 2008). In enhancing the ameliorative impact of metformin in DCM, curcumin down-regulates the expression of TGF-β1 via inhibition of JAK/STAT signaling pathway, leading to reducing inflammation and improving DCM (Abdelsamia et al., 2019). Cardiac fibrosis is a common phenomenon during DCM. It has been demonstrated that enhanced accumulation of ECM commonly occurs in cardiac fibrosis. The collagen type I and III are main elements of ECM (Abdel et al., 2012; Russo and Frangogiannis, 2016). So, reducing the level of these components can pave the road into cardiac fibrosis treatment during ECM. The *in vivo* experiment on animal model of DM (rat) demonstrates that curcumin administration (300 mg/kg) for 16 weeks improves cardiac fibrosis via decreasing accumulation of collagen type I and II in ECM. The investigation of molecular pathways reveals that in attenuation of cardiac fibrosis, curcumin down-regulates TGF-β1, TβRII and Smad2/3, while it induces Smad7 expression (Guo et al., 2018).

### Infection

The *candida albicans* is a commensal yeast of genital and intestinal tracts. The increasing evidence has shown that *candida albicans* is a pathogenic yeast in women and can induce vulvovaginal candidiasis (VVC) in the presence of other diseases such as DM and immune disorders (Deorukhkar and Saini, 2013). In respect to immunomodulatory impact of curcumin, its administration can be beneficial in VVC treatment. By reducing the level of IL-1β (pro-inflammatory factor) compared to TGF-β (anti-inflammatory factor), an amelioration occurs in VVC and paves the road for efficient treatment of this infection (Rodero et al., 2018).

## Clinical Studies

Nowadays, we are witnessing that a high number of studies evaluate the efficiency of drugs in both *in vitro* and *in vivo* experiments. However, clinical translation of these studies is of importance in directing into commercial application. Notably, the effect of curcumin on TGF-β level has been evaluated in clinical trials (Panahi et al., 2016). In this study, 117 patients were enrolled and they were randomly divided into two groups including placebo (n = 58) and treatment (n = 59). The treatment group received curcumin daily at the dose of 1 g for 8 weeks. The results of this study revealed that curcumin is advantageous in treatment of metabolic syndrome. This plant derived-natural compound is able to diminish serum levels of pro-inflammatory cytokines, and among them, TGF-β level demonstrates a remarkable decrease after curcumin supplementation, showing the potential of curcumin in treatment of metabolic syndrome. The oral squamous cell carcinoma (OSCC) is one of the malignancies affecting high number of people worldwide (Saranath et al., 1999). A variety of factors contribute to OSCC development, and among them, oral submucous fibrosis (OSMF) is of importance (Lippman and Hong, 2001; Reibel, 2003). The stimulation of inflammation by myofibroblasts enhances levels of TGF-β that subsequently, promotes deposition and generation of ECM (Khan et al., 2012). A pilot study has been performed on 28 patients (23 males and five females) to evaluate the efficiency of curcumin in decreasing TGF-β levels. The treatment group received a mixture of curcumin and piperine (300 mg) twice daily in a period of 9 months. The findings revealed that curcumin is able to considerably diminish TGF-β expression by 32.1% (Gupta et al., 2017), showing that curcumin can be applied as a potent chemopreventive agent.

**FIGURE 3:**
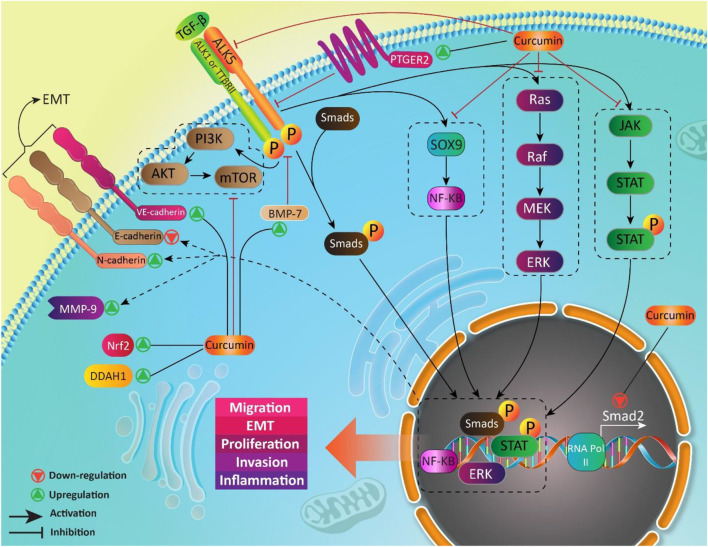
TGF-β as a down-stream target of curcumin in different diseases.

**FIGURE 4:**
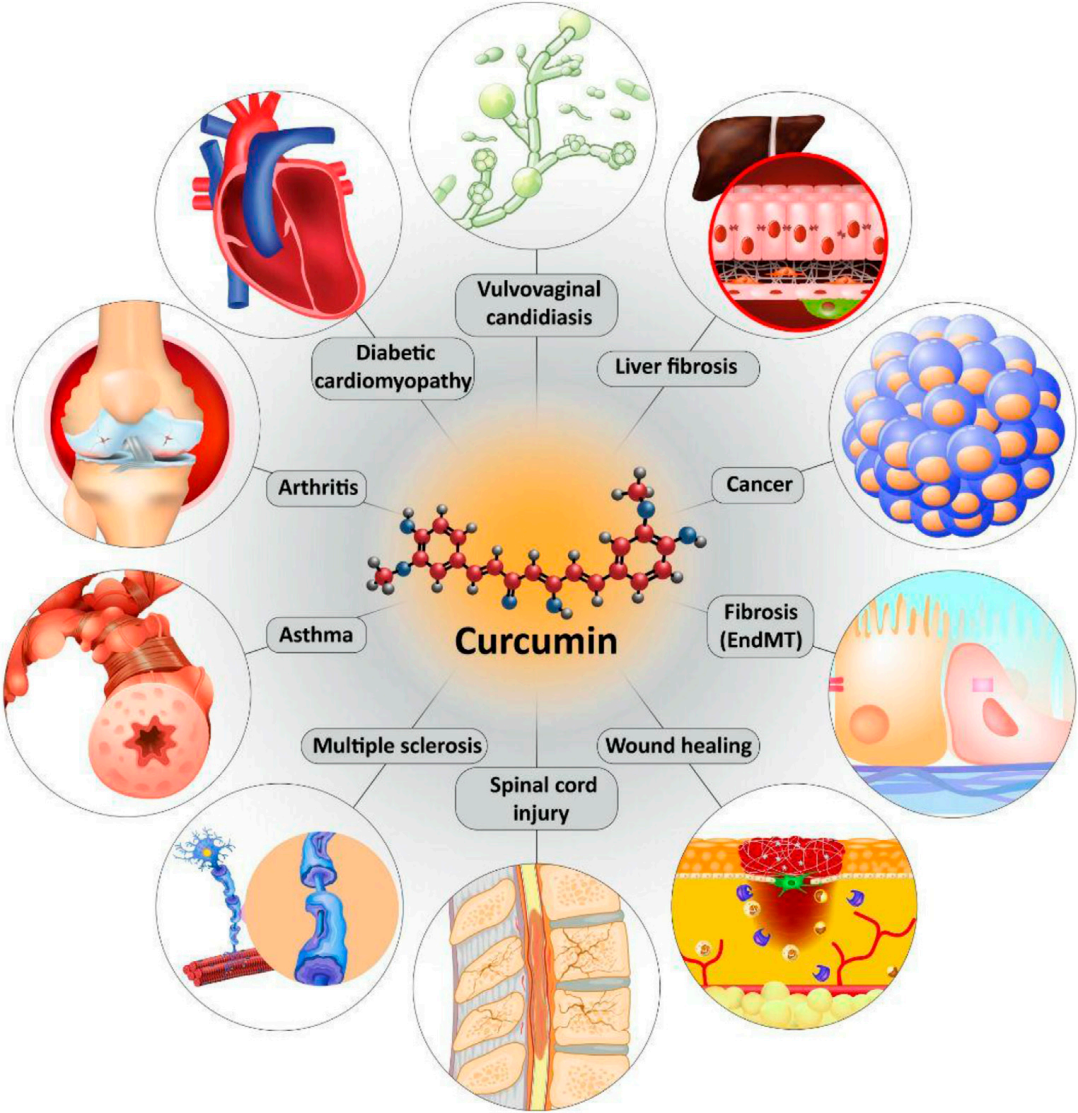
The protective effects of curcumin in different diseases mediated by its effect on TGF-β signaling pathway.

We previously discussed curcumin and its effect on TGF-β in MS treatment. Regulatory T cells (Treg cells) are key players in MS. Normally, Treg cells contribute to self-tolerance preservation and regulation of immune responses against infections and cancer cells (Fujio et al., 2010). Treg cells are capable of secretion of TGF-β. Noteworthy, TGF-β is vital for differentiation of Treg cells (Sakaguchi and Sakaguchi, 2005). In preventing inflammation and immune responses, Treg cells secrete TGF-β (Wan and Flavell, 2008). So, there is a dual relationship between Treg cells and TGF-β, so that Treg cells exert their anti-inflammatory action through TGF-β secrion, and also, TGF-β is necessary for Treg cell differentiation. Any impairment in this interaction can predispose to development of inflammatory diseases such as MS. Recently, nanocurcumin has been developed for treatment of MS patients. As curcumin suffers from poor bioavailability, loading it on nanoparticles promotes its therapeutic effects. Nanocurcumin administration significantly enhances TGF-β expression and also, its secretion levels. Based on interaction between TGF-β and Treg cells, enhanced expression and secretion of TGF-β by nanocurcumin result in an improvement in function of Treg cells, and alleviation of MS (Dolati et al., 2019). Table 1 summarizes the therapeutic effects of curcumin mediated by its effect of TGF-β. Figures 3 and 4 summarize the therapeutic impacts of curcumin mediated by its effect on TGF-β signaling pathway.

**TABLE 1:** The modulatory effect of curcumin on TGF-β in different diseases.

Disease/protective effect	Cell line	*In vitro*/*in vivo*	Dose	Duration of experiment	Administration route	Outcomes	Refs
Anti-inflammatory	Peripheral blood mononuclear cells	*In vitro*	10 μM	1 h	–	Reducing the expression of TGF-β to suppress lipopolysaccharide-mediated inflammation	Vasanthkumar et al. (2019)
Anti-inflammatory	–	*In vivo*	0, 196.11, 393.67, 591.46 and 788.52 mg/kg	60 days	Diet	Curcumin exerts anti-inflammatory activity by down-regulation of TGF-β1	Ming et al. (2020)
Anti-inflammatory	Mouse macrophage cells Raw246.7	*In vitro*	5, 10, 50, 100, 200 and 500 μg/ml	24 h	–	A combination of curcumin and curcumol exerts anti-inflammatory activity by decreasing the release of fibrotic factors by inhibiting Smad2/3 phosphorylation through TGF-β down-regulation	Godwin et al. (2019)
Anti-inflammatory	–	*In vivo* (animal model of airway inflammation)	10 and 20 mg/kg	1–72 h	Intraperitoneal	Improving airway inflammation via down-regulation of TGF-β1	Kumari et al. (2017)
Anti-inflammatory	–	*In vivo* (experimental model of colitis)	100 mg/kg/daily	24 h after colitis induction	Intragastrically	Improving colitis by enhancing the level of anti-inflammatory cytokine TGF-β1	Zhao et al. (2017)
Anti-inflammatory	–	*In vivo* (animal model of colitis)	100 mg/kg	7 days	Oral gavage	Amelioration of colonic weight, histopathological scores, and remitting pathological injury via down-regulation of TGF-β1	Zhao et al. (2016)
Anti-inflammatory	Sertoli cell line 93RS2	*In vitro*	50 and 100 μM	6 h	–	Inhibiting the stimulatory impact of gallic acid on TGF-β1 to abate inflammation and improve reproductive system	Abarikwu et al. (2014)
Multiple sclerosis	–	*In vivo* (animal model of autoimmune encephalomyelitis)	20 mg/kg	21 days	Intraperitoneal	Upregulation of TGF-β1 acts as an anti-inflammation factor	Esmaeilzadeh et al. (2019)
Anti-fibrotic	–	*In vivo* (paraquat-challenged rats)	200 mg/kg	14 days	Intraperitoneal	Improving pulmonary function and inhibition of fibrosis via down-regulation of TGF-β1	Chen et al. (2017)
Anti-fibrotic	IEC-6 cells	*In vitro*	2.5, 5 and 10 μM	24 h	–	Alleviation of intestinal fibrosis by inhibition of EMT via down-regulation of TGF-β1 and inhibition of smad phosphorylation	Xu et al. (2017)
Anti-fibrotic	Mouse lung fibroblast cell line	*In vitro*	50 μM	0, 24, 48 and 72 h	–	Attenuation of pulmonary fibrosis by down-regulation of TGF-β2 and subsequent inhibition of fibroblast differentiation into myofibroblasts	Liu et al. (2016a)
Anti-fibrotic	Rat mesangial cells	*In vitro* in vivo (animal model of diabetes)	−10 mg/kg/day	48 h 56 days	Intraperitoneal	Improving renal fibrosis in diabetic rats by down-regulation of TGF-β1 and reducing ECM accumulation	Ho et al. (2016)
Anti-fibrotic	–	*In vivo* (animal model of cardiac fibrosis)	150 and 300 mg/kg/day	28 days	Oral gavage	Suppressing myofibroblast differentiation and alleviation of cardiac fibrosis via down-regulation of TGF-β1	Ma et al. (2017)
Anti-fibrotic	Rat normal renal interstitial fibroblast cells (NRK- F4^9^F) and rat normal renal tubular epithelial cells (NRK-52 E)	*In vivo* (animal model of renal fibrosis)	10, 20 and 30 μM 50 and 100 mg/kg	24 h 14 days	Gastro gavage	Inhibition of local fibroblast proliferation and reducing ECM deposition via down-regulation of TGF-β1/Smad2/3	Zhou et al. (2014)
Anti-fibrotic	Cardiac fibroblasts	*In vitro*	0, 5, 10 and 20 μmol/L	24 h	–	Inhibiting fibroblasts differentiation and alleviation of cardiac fibrosis via down-regulation of TGF-β1 and its down-stream targets Smad2 and MAPK	Liu et al. (2016b)
Anti-fibrotic	Human circulating fibrocytes	*In vitro*	20 μM	72 h	–	Decreasing TGF-β1 expression is related to reduced migration and differentiation of fibroblasts and subsequent amelioration of fibrosis	Fu et al. (2015)
Anti-fibrotic	Cardiac fibroblasts	*In vitro*	25 μΜ	24 h	–	Inhibition of ECM deposition and suppressing migration and proliferation of cardiac fibroblasts via down-regulation of Smad2/3, leading to attenuation of fibrosis	Chung et al. (2014)
Cancer	BCPAP cell line, derived from a human papillary thyroid carcinoma	*In vitro*	12.5, 25 and 50 μM	24 h	–	Exerting anti-metastatic activity by suppressing TGF-β signaling pathway, inhibiting Smad2/3 interaction with Smad4, and their nuclear translocation, leading to a diminution in EMT	Zhang et al. (2016)
Cancer	MCF-7 cell line	*In vitro*	15 μM	24 and 48 h	–	Suppressing angiogenesis and metastasis of cancer cells by down-regulation of TGF-β, as an upstream modulator of NF-κB signaling pathway	Mohankumar et al. (2015)
Cancer	–	*In vivo* (animal model of hepatocellular carcinogenesis)	100 mg/kg	15 days	Orally	Suppressing cancer carcinogenesis by down-regulation of TGF-β, leading to inhibition of angiogenesis and induction of apoptosis	Abouzied et al. (2015)
Cancer	MDA-MB-468, MDA-MB231, BT-549, and BT-20 cells	*In vitro*	20 μM	48 h	–	Suppressing doxorubicin-mediated EMT and metastasis of triple negative breast cancer cells via inhibition of TGF-β1	Chen et al. (2013)
Cancer	Colon carcinoma cells HCT116 and MRC-5 fibroblasts	*In vitro*	5 μM	4 h	–	The interaction between cancer cells and fibroblasts enhances the expression of TGF-β3/Smad2 to induce EMT. Administration of curcumin reverses this axis	Buhrmann et al. (2014)
Liver diseases	–	*In vivo* (animal model of liver cirrhosis)	400 mg/kg	7 weeks	Oral route	Improving liver cirrhosis and protection against thioacetamide-mediated liver injury via inhibition of TGF-β1 expression	Eatemadi et al. (2014)
Hepatoprotective	–	*In vivo* (animal model of bile duct ligation)	100 mg/kg	4 weeks	Oral gavage	Improving hepatic fibrosis partially by reducing the expression of TGF-β1	Eshaghian et al. (2018)
Liver injury	Human LO2 hepatocytes	*In vitro*	5, 10, 20, 30, 50, 70 and 100 μM	72 h	–	Inhibition of cobalt chloride-mediated liver injury by suppressing EMT via suppressing TGF-β/Smad2/3 axis	Kong et al. (2015)
Hepatotoxicity nephrotoxicity	–	*In vivo* (animal model of lead toxicity)	200 mg/kg	4 weeks	Oral route	Alleviation of liver and kidney injuries by down-regulation of TGF-β1	Soliman et al. (2015)
Renoprotective	–	*In vivo* (animal model of diabetes)	0.2 mg/kg	8 weeks	Oral gavage	Protecting kidney of DM rats against inflammation and fibrosis via down-regulation of TGF-β1	Liu et al. (2017)
Osteoprotective	–	*In vivo* (animal model of periodontitis)	400 mg/kg	15 days	Oral gavage	Improving collagen and bone repairs via upregulation of TGF-β	Guimaraes-Stabili et al. (2019)
Anti-asthmatic	–	*In vivo* (animal model of bacterial-mediated asthma)	2.5 ml at 10 mg/kg	2 h before antigen challenge	Intranasal	Inhibition of lipopolysaccharide-mediated asthma partially by reducing expression of TGF-β	Kumari et al. (2019)
Neuroprotective	-	*In vivo* (rat model of spinal cord injury)	30, 100 and 300 mg/kg	7 days	Intraperitoneally	Improving neural function by inhibition of both TGF-β1 and TGF-β2 and reducing ECM accumulation	Yuan et al. (2015)
Gingival overgrowth	Fibroblasts	*In vitro*	0, 1, 5, 10, 15 and 20 μM	12 h	–	Curcumin interferes with gingival overgrowth by inhibition of TGF-β1-mediated connective tissue growth factor induction via suppressing Smad2	Chen et al. (2018)
Wound healing	-	*In vivo* (diabetic rat)	400 μL	19 days	Topically	Enhancing the expression of TGF-β1 on days 3, 7 and 14 to induce angiogenesis, resulting in accelerating wound healing	Kant et al. (2015)
Benign prostatic hyperplasia	–	*In vivo* (male wistar rats)	50 mg/kg	4 weeks	Oral route	Amelioration of benign prostatic hyperplasia by reducing expression of TGF-β1, leading to decreased prostate volume	Kim et al. (2015)
Sepsis	–	*In vivo* (rat model of sepsis)	10 and 20 mg/kg	–	Intraperitoneal	Protection against lipopolysaccharide-mediated vasoconstriction dysfunction by down-regulation of TGF-β1	Lu et al. (2015)

## Conclusion and Remarks

Curcumin is a naturally occurring nutraceutical compound with excellent therapeutic and biological activities. A look at PubMed demonstrates that annually, a high number of studies investigate the protective effects of curcumin against various diseases with a focus on underlying molecular pathways. In the present review, we comprehensively discussed the role of TGF-β in protective effects of curcumin. Noteworthy, curcumin both upregulates/down-regulates TGF-β signaling pathway in diseases therapy. The most studied therapeutic effect of curcumin mediated by TGF-β regulation is anti-fibrotic. Different studies have shown that curcumin inhibits migration and proliferation of fibroblasts and their differentiation by down-regulation of TGF-β. Curcumin inhibits EMT by suppressing TGF-β to ameliorate collagen synthesis and cell migration during fibrosis. It is worth mentioning that curcumin inhibits chemotherapy-mediated fibrosis via down-regulation of TGF-β. In alleviation of fibrosis, curcumin reduces ECM deposition and accumulation by targeting TGF-β. The interesting point is that curcumin inhibits EMT-mediated fibrosis. In this way, curcumin is able to target down-stream mediators of TGF-β such as PI3K/Akt/mTOR pathway. Another potential therapeutic effect of curcumin mediated by its effect of TGF-β is anti-tumor activity. In spite of great advances in medicine, cancer is still a big challenge for scientists. Curcumin exerts anti-metastatic activity via inhibition of TGF-β-mediated EMT. It also inhibits progression of cancer cells by suppressing CAFs via TGF-β down-regulation. By inhibition of TGF-β, curcumin protects liver cells against toxic agents. In neurological disorders and arthritis as well as asthma, curcumin exerts anti-inflammatory activity via targeting TGF-β signaling pathway. In accelerating wound healing, curcumin inhibits TGF-β1/Smad7 axis, while it induces TGF-β3. It is held that using nanoparticles enhances bioavailability and capability of curcumin in affecting TGF-β signaling pathway. In diabetes, fibrosis is a common phenomenon due to increased accumulation of collagen type I and III that is inhibited by curcumin via down-regulation of TGF-β. The important point is that clinical trials have shown that efficacy of curcumin in regulation of TGF-β in treatment of metabolic syndrome. All of the studies are in line with modulatory impact of curcumin on TGF-β in different diseases. However, more studies are required to clarify mentioned discussions.

## Author Contributions

HM and MN contributed in conception, design, statistical analysis and drafting of the manuscript. MA, AZ, KH, FH, VZ, ERM, SS, and PM contributed in data collection and manuscript drafting. All authors approved the final version for submission.

## Conflict of Interest

The authors declare that the research was conducted in the absence of any commercial or financial relationships that could be construed as a potential conflict of interest.

## Glossary

TGF-β, transforming growth factor-β;
DMC, demethoxycurcumin;
BDMC, bis-demethoxycurcumin;
Nrf2, nuclear factor erythroid 2-related factor 2;
HO-1, heme oxygenase-1;
SOD, superoxide dismutase;
NQO1, NADPH quinone reductase 1;
DM, diabetes mellitus;
CAVD, calcified aortic valve disease;
NF-κB, nuclear factor-kappaB;
ER, endoplasmic reticulum;
GRP78, glucose-regulated protein 78;
CHOP, CCAAT-enhancer-binding protein homologous protein;
ATF4, activating transcription factor 4;
UPR, unfolded protein response;
BPA, bisphenol A;
LAP, latency-associated peptide;
TβRII, TGF-β type II;
ALK5, TGF-β type I;
TGIF, tumor growth-interacting factor;
I-Smads, inhibitor Smads;
MAPK, mitogen-activated protein kinase;
PI3K, phosphatidylinositol 3-kinase;
FAPs, fibroadipogenic progenitors;
TRIM33, tripartite motif-containing 33;
ECM, extracellular matrix;
miR, microRNA;
EMT, epithelial-to-mesenchymal transition;
Brb, berberine; Res, resveratrol;
CCl_4_, carbon tetrachloride;
TIME, carbon tetrachloride;
TME, tumor microenvironment;
CAFs, cancer-associated fibroblasts;
MMPs, matrix metalloproteinases;
PQ, paraquat;
OX, oxaliplatin;
PTHrP, peptide parathyroid hormone-related protein;
BMP-7, bone morphogenic protein-7;
EndMT, endothelial-to-mesenchymal transition;
ADMA, asymmetric dimethylarginine;
DDAH1, dimethylaminohydrolase-1;
NO, nitric oxide;
NOS, nitric oxide synthase;
CP, cisplatin;
ATO, arsenic trioxide;
PD, peritoneal dialysis;
ESRD, end-stage renal disease;
PGE2, prostaglandin E2;
PTGER2, prostaglandin E receptor 2;
IPF, idiopathic pulmonary fibrosis;
PVR, proliferative vitroretinopathy;
RPE, retinal pigment epithelial;
EGCG, epigallocatechin gallate;
MS, multiple sclerosis;
CNS, central nervous system;
SCI, spinal cord injury;
LIDD, lumbar intervertebral disc degeneration;
MH_2_, MAD homolog 2;
SARA, Smad anchor for receptor activation,
CNs, curcumin-nanoparticles;
RA, rheumatoid arthritis;
DCM, diabetic cardiomyopathy;
DMI, DM type I,
DMII, DM type II;
JAK/STAT, Janus kinase/signal transducer and activator of transcription;
VVC, vulvovaginal candidiasis;
OSCC, oral squamous cell carcinoma;
OSMF, oral submucous fibrosis.
